# Father Involvement in Early Childhood Care: Insights From a MEL System in a Behavior Change Intervention Among Rural Indian Parents

**DOI:** 10.3389/fpubh.2020.00516

**Published:** 2020-09-30

**Authors:** Sapna Nair, Shivani Chandramohan, Nandhini Sundaravathanam, Arvind Balaji Rajasekaran, Rathish Sekhar

**Affiliations:** ^1^Institute for Financial Management and Research, LEAD at Krea University, Chennai, India; ^2^Emory University Rollins School of Public Health, Atlanta, GA, United States

**Keywords:** child development, father involvement in childcare, monitoring, evaluation and learning (MEL), measurement for change, norm change, early childhood development (ECD)

## Abstract

**Introduction:** Fathers' involvement in care and early initiation of cognitive development activities have a positive impact on a child's social-behavioral, cognitive-academic and emotional-psychological development. This research study, conducted in Tamil Nadu in south India (2017–19), employed a Cluster Randomized Trial to test the impact of techno-social innovations in improving the involvement of fathers in child-care on child development outcomes. Qualitative studies were used to inform the trial and provide insights into pathways of change.

**Objective:** This paper discusses the design, implementation and results of the study through the monitoring, evaluation and learning (MEL) framework to provide an understanding of the perceptions among parents and service providers surrounding early child development, the adaptations and learnings through the intervention period, and changes that were brought about through the intervention.

**Methods:** The study was at a Proof of Concept stage, and the primary learning objective was to keep the learning process going through the period of the study, as well as obtain evidence to inform future model development. The measurement for change process in the study occurred in three distinct yet interconnected stages. In the first stage, the program was planned, and the design was refined for both the implementation and evaluation of the project. The next stage was the actual implementation: with a learning loop during the execution of the main intervention. The third stage was intended to reflect on the adaptations and pathways to change through the project period and collate evidence for model refinement.

**Results and Discussion:** The data collected from the formative research was used to design, develop and implement the intervention. Lessons in coordination with the government program not only brought policy visibility, access to secondary data, and enabled field research, but also provided access to a workforce with immense field knowledge and presence in the rural underserved population. In order to continuously inform the implementation process of the intervention, the feedback loops allowed for adaptions to be made at each stage. The findings provide insights for programming early childhood development interventions, especially interventions regarding improving father's involvement in child-care, and ways to leverage evidence in these interventions.

## Introduction

The first 3 years of a child's life is a period when substantial brain development occurs, and, therefore, this is a crucial time to build children's capacities through experiences and interactions (1, 2). Higher involvement of fathers in parenting has a role in improving all forms of human capacities, including cognitive-academic (3–6), social-behavioral (5, 6) and emotional-psychological development (7). However, in many countries, mothers remain as the predominant caretakers. Work continues to be the dominant part of a man's life, and the role of a father as a caregiver is seen as ancillary. Such negative perceptions toward fathers' involvement can also be fostered by mothers, a concept known as Maternal Gatekeeping (8), or by child development service providers, largely known as a “Culture of Maternalization.”

Fathers' involvement is a multidimensional concept (9, 10), with an interplay of several factors like parental relationship, physical presence, residential/non-residential fathers and socio-economic factors (11). A major reason for the minimal participation of men in child-care is deeply ingrained traditional gender roles as well as norms and responsibilities, which pardon absent or uninvolved fathers (12). Often, fathers tend to play with the child rather than involve in higher degree care activities that “undo gender norms,” such as cooking and bathing the child (13). Fathers may find it hard to keep up with the changing requirements of the baby due to their work roles—while mothers are presumed to be naturally knowledgeable on parenting (14, 15). In order to modify behaviors, it is essential to understand the origins of particular beliefs as local norms often constitute barriers to change (16). By targeting key factors, father involvement and the resulting outcomes can be modified to promote the desired behaviors—resulting in norm change.

The Integrated Child Development Services program (ICDS), is the flagship child development scheme of the Government of India. For children within the 0–3 years' age group, the program focuses on cure, nutrition, and prevention, and is yet to integrate aspects of cognitive development. The program also hinges on the mother as the primary recipient of information and action. There has been no interventional research to evaluate the impact of fathers' involvement in child-care, and the resulting child developmental outcomes in the Indian context. Some studies demonstrate the patriarchal norms that restrict the nature of fathers' involvement (10) and deep-rooted attitudes and perceptions toward involvement (17).

The existing ICDS framework was supplemented with gender approaches and a techno-social innovation, with the intention to increase the involvement of fathers in child-care and evaluate the impact on child development. The intervention took place for 1 year and was incorporated into the ICDS outreach in Tamil Nadu. Beneficiaries of the intervention consisted of 700 children in the 0–3 years' age group and 1,400 parents predominately from low socio-economic status (SES) groups, as well as 57 Anganwadi workers (AWWs), government-based community workers, belonging to the identified Anganwadi centers (AWC), or public crèches, from the districts of Madurai and Dindigul in Tamil Nadu. There were two major components of the intervention strategy. One: using information technology to disseminate information to parents through the network of AWWs. This intervention was implemented through in-person outreach, which was strengthened through the use of computer tablets with an application called *Arivu* to provide tailored information, as well as electronic outreach through text messages on mobile-phones (Short Message Services, SMS) which provided targeted information on early stimulation, parenting, the involvement of fathers, and nutrition. Two: increasing the involvement of fathers in child-care by conducting regular fathers group meetings which involve selected father role models, using a calendar maintained by the mother, and providing incentives in the form of mobile phone credit.

Since this is a proof of concept study, and there are no studies addressing fathers' involvement in India, the study employed an initial exploratory phase before the trial. The aim was to explore factors that influence fathers' involvement in order to detail the implementation plan, monitor and document the implementation, and finally gather evidence of the impact through a rigorous trial. The monitoring, evaluation, and learning (MEL) occurred in three rounds: the primary round to develop the intervention, the second round to facilitate the intervention and ensure it runs effectively, and the final round of learnings to inform future model development in India. The results presented in this paper discuss the MEL system implemented in this intervention, along with the analysis of qualitative research methods adopted as a part of the impact study. Therefore, this paper seeks to understand how the climate of rural India's cultural, economic, and social beliefs shape paternal involvement in early childhood care among rural parents, how father-child dynamics have altered throughout the process, and how to strengthen father involvement interventions in the child development space.

## Materials and Methods

The measurement for change in this study occurred in three distinct yet interconnected stages. In the first stage, the program was planned, and the design was refined for both the implementation as well as the evaluation of the project. In the second stage, there was the actual implementation: with a learning loop during the execution of the main intervention, and the evaluation itself, which was left intact as it was a cluster randomized trial. The third stage was to reflect on the adaptations and pathways to change through the project period, and collate evidence for model refinement. The methods and processes adopted in each of the stages were as follows.

### Stage I: Planning and Design

**Figure 1:**
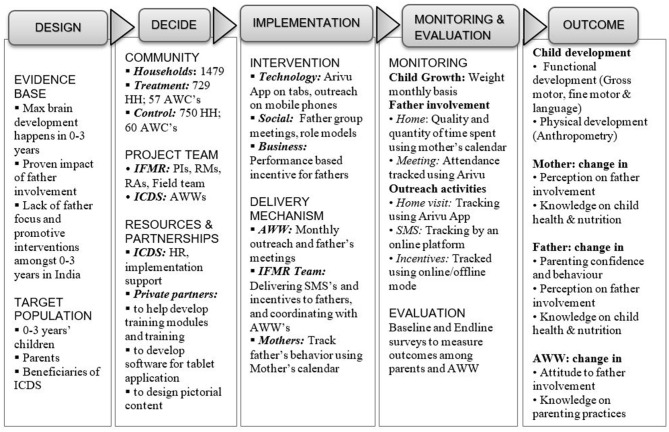
Theory of change developed at the beginning of the project implementation.

The main study was designed earlier, and was peer reviewed for funding by Grand Challenges Canada led by the Canadian Institutes of Health Research in December 2016. In the planning and design stage there were two main learning objectives. One was to arrive at strategies to design the implementation plan. Two was the conceptual framework of the importance of father involvement in child-care, and how behavior change can happen through the adoption of norm changing communication. Programming was then developed accordingly through literature review and study of other successful models. At this point the conceptual framework and the implementation framework (Figure 1) were to be refined with evidence from the ground.

#### Formative Study

The implementation model was initially refined through learnings generated from a formative qualitative study with the beneficiary groups in the intervention sites. The formative study was conducted in the two intervention districts, Madurai and Dindigul. Semi-structured interviews, Focus Group Discussions (FGD) and Key Informant Interviews (KII) were conducted with fathers, mothers, AWWs, and the district ICDS team over a period of 3 months from June to August 2017. KII were also conducted initially with the state heads of ICDS. These interviews along with the literature review and previous knowledge on the topic, led to the development of the initial categories and guidelines for the intervention modules.

#### Secondary Data Analysis

Secondary data available from the state ICDS department pertaining to the number of children and households available in the Madurai and Dindigul districts, the GIS coordinates for the AWC, and the staffing plan for the centers in the two districts were analyzed. The analysis of these three data sets was used to inform the project implementation plan and the intervention design. Secondary data analysis, including analysis of all the protocols in the state behavior change guidelines and communication products such as media, was conducted in order to inform content development for the intervention.

### Stage II: Implementation of the Project

The project was implemented for 1 year (January to December 2018) in rural Tamil Nadu through the existing program of the state ICDS department. The impact evaluation was not a focus for change in itself, as no changes could be made to the intervention or control arms during the intervention to avoid any biases in the trial. Measurements for change and the learning loop, to incorporate evidence during this stage, was limited to the implementation of the intervention itself: core program monitoring for outputs and activities, and evidence on changes in some early secondary outcomes. As an ongoing process secondary data, guidelines, and discussions with the state program implementation was maintained in order to ensure congruence with the broader public program, in which the project was embedded in, and to ensure policy uptake. For this monthly implementation, updates were provided to the ICDS at the state and district levels.

#### Program Monitoring

Given that there were three strategies in intervention to reach the beneficiaries, our monitoring mechanism aimed to collect and analyze information regarding the efficacy of each of these strategies. Emergent learnings from the field from each strategy were used to innovate overcome challenges or improve the strategy in a consensual manner with discussions with the AWW in the districts, district program staff of ICDS, the project implementation staff and the research team, incorporating changes either in monthly meetings or immediately afterwards. Information was received using a combination of online data collection and monitoring supplemented by field level (on-ground) monitoring.

##### a) Technology innovation: tablet-based information sharing and outreach

The objective of monitoring was to understand how the tablet-based outreach is happening periodically and the quality of the outreach. This was done using data from the app, including GIS entries and monthly reviews on the process with AWW. There was also a process of triangulation with the entries of the mothers in the calendars.

##### b) Group mobilization: monthly father group meetings

The objective of the monitoring exercise was to document participation and use the information to ensure high participation in monthly father group meetings at the AWC. For collecting meeting status, the Anganwadi worker is required to validate, using the tablet application, whether the meeting was conducted on a given month and also record the participation of the fathers who are part of the program. This data was analyzed every month for coverage. To ensure quality, the project team and the AWW visited households together to understand why certain fathers were absent in the meeting, and if the schedule and content were acceptable.

##### c) Business innovation: performance-based mobile recharges for fathers

The objective here was to ensure that disbursement of incentives to fathers is happening at the planned intervals, and all fathers were receiving their monthly performance incentives. For this objective, the success rate of mobile recharges was analyzed on a monthly basis using quantitative data on monthly recharge status from the online platform used to disburse the recharges through an automatic process. This data was substantiated using random phone calls to verify and understand the causes of non-receipt.

#### Following Up on Secondary Intermediate Outcomes

The quality of fathers' involvement was captured through calendars by the mothers every month and these were collected by AWWs and digitized by project staff. The calendars provided a real-time update of fathers' involvement with their child in a household: through a quantitative scale as an objective measure of the time spent by the father, and a qualitative scale on the mother's perception of the quality of the time spent. This information was collated and analyzed every month.

#### Evaluation Trial

The evaluation of the impact of the project was performed through a Cluster Randomized Control Trial. Quantitative measurements were analyzed through a comparison of baseline and endline measures in intervention and control arms for the following indicators: child development metrics and anthropometrics, attitude and behavior related to fathers' involvement in child development activities among the parents, and knowledge on child development and attitude toward involving fathers in child-care among the community workers. A longitudinal case study among twenty families was planned, in order to observe changes in the family environment and understand processes of change.

### Stage III: Model Refinement

For capturing learnings, refining the model for scale-up, and informing new model development, the objective was to document and collate what worked and how at the end of the project period. The evidence from the implementation strategies was collated using qualitative methods. Though the evidence from the impact evaluation was available and included insights from the qualitative studies, this was retained as a separate evidence piece to establish the impact of the interventions on primary child development outcomes. The evidence here pertains to program implementation and beneficiary engagement.

The longitudinal case studies from seven families were analyzed in an interpretive thematic exploration to look at SES of families, education and job profile of parents, availability of care support, and aspects of father involvement. Father involvement has two aspects; one, scoring of father involvement from the quantitative surveys based upon participation in physical-biological, cognitive and socio-emotional caregiving. Two, the frequency and consistency of father involvement, measured by quantity and quality of time spent, from the mother's calendar. These were interpreted against development gains of the child, in the year of the intervention. By the endline, data was available from seven families. Focus group discussions were conducted with fathers, mothers and AWW in March 2019 during the endline data collection.

The implementation framework was made operational through designing the implementation plan, including the monthly plans for father's meetings, outreach content and guidelines. The business model to provide incentives to fathers was also planned. In keeping with the ethical treatment of subjects, at the end of the study, the control group was given the information available through the study. Workshops were conducted with the parents and AWWs in the control areas to provide the information on the modules on child development, cognitive development, child-care and parenting. Pamphlets, printed mobile messages, activities conducted during the fathers' group meetings, and any other study material developed for the intervention were made available to the control group through the AWWs of these areas. The study protocol and tools, including consent processes, were reviewed and approved by IFMR Human Subjects Committee (dated September 26, 2017). Written informed consent to participate in the study was provided by all parents who participated in the evaluation study and who were a part of the intervention. The formative study on general norms on parenting in the villages was done with informed oral consent. The parents for the FGDs were invited to the discussion after they were informed in advance about the purpose of the study. We also had informed oral consent for KIIs with the government officials who are our intervention partners.

## Results

The data collected from the formative and qualitative work was used to design, develop and implement the intervention. In order to continuously inform the implementation process of the intervention, the feedback loops allowed for adaptions to be made at each stage.

### Stage I

#### Partnership Development

Across the hierarchy, there was engagement from the very beginning with officials and workers from the ICDS Department, which is responsible for running the Anganwadi centers in the state. Close coordination with the government program not only brought policy visibility and reach into the community, but it also enabled access to secondary data and information. The partnership enabled formative studies to be conducted, piloting of various tools, and completion of the surveys.

#### Learnings

The engagement had formal procedures to seek inputs and data, and reporting channels through emails, presentations and submission of monthly reports in hard and soft copies. While these procedures added some amount of administrative burden, they also afforded access to the immense field knowledge and presence of the ICDS Department, which is crucial in reaching out to the rural underserved population in India. The relationship had to be built on a personal basis at every level and had to be reinforced when each official was transferred. The coordination also had to be particularly responsive at the district and block level to implement the field plans effectively.

#### Formative Studies

The formative assessment contributed to the MEL process by deepening an understanding of the community and its prevailing norms. In order to facilitate norm change, it is essential that along with understanding the innate and intrinsic norms of a community, the factors and facilitators of change are identified (16). The important takeaways from the research that were useful for designing the intervention were regarding three aspects:

##### Fathers are important figureheads

Fathers were perceived to be important as figureheads, and practical aspects of their involvement with the care of young babies is not the prevailing cultural norm. Fathers did not consider it their duty to take care of their child or participate in daily caregiving chores and were of the view that their time after work should be dedicated to de-stressing instead of taking care of children.

Mothers comply with this notion and believe that disturbing fathers may affect the household income. Some fathers even expressed difficulty in admitting they helped with the child. This is strongly indicative of the societal pressure felt by fathers to adhere to established gender norms.

“*As a father, my main responsibility is to earn for the family so I cannot do all the work a woman does. Activities like feeding, bathing, healthcare, and cleaning are taken care of by my wife since she is at home and has more time. This is how we were raised.” (Father)*

Fathers' occupations often kept them away from the family and the child for many hours in the day. However, very few families identified this as the reason for the high involvement of the mother in their child's lives. The division seems to be motivated by gender norms more than its economic value. Fathers are expected to be busy in earning for the household, and not get involved with their child's day-to-day needs.

We are following the same practices we have seen and learned from our elders/parents and follow societal guidelines; maybe as per the economic conditions the products we give to our kids may vary, but otherwise, it is all the same. (Mother)

The women primarily take care of the home and child or engage in part-time work. Due to this, they have much more time to devote to child-care duties. The importance of the father's role as provider and breadwinner overshadowed their role in providing care in that there was not only less responsibility in taking care of children, but also entitlement for rest hours. These were all prevalent norms in the community that needed to be altered through the intervention, as a means of promoting father involvement.

##### Learnings

The modules and the Arivu App encouraged the idea of the family as a unit for providing care for the child. Given that the prosperity and better future of the family was a common goal, both parents should be contributors to the development of their child. All fathers wanted to provide their children with a better future and were willing to make the necessary changes in order to improve their educational outcomes. Therefore, norm change was targeted by using education as a motivational factor. However, fathers initially prioritized that their child become a doctor or engineer, and this was another aspect that was factored into the modules. The modules incorporated lessons on how parents need to be proud of their child's achievements regardless of the path they take. Some fathers felt that incentives for involvement were unnecessary as it was their duty to provide for the kid. Thus, the component of incentives was introduced more as a way to encourage fathers and for them to monitor their own change in involvement, and therefore was kept as a minimal amount of money.

##### Health and safety as care for young babies

For young babies, child-care needs were perceived to encompass meeting health and nutrition needs, as well as ensuring safety. Engagement with the child and encouragement for child development and growth activities were not perceived as important aspects of the care domain.

“*At this age, a child needs complete monitoring and attention for each and every need. This depends on the parents, as they have to be careful and watch the child always. The children do not know what is good or bad, what to eat and what not to eat, and while playing someone should definitely be here to watch them and ensure they don't get hurt.” (Father)*

When asked to identify the basic needs of a child, parents primarily focused on healthy food, cleanliness, and affection.

“*Children depend on us for all of their needs, and it is our responsibility to provide them with a prosperous life. Food, healthcare, and teaching good habits are the most important things that need to be taken care of at this time.” (Mother)*

There was a general perception that parents in the current generation are much more involved in the parenting process, as compared to the previous generation. Since the structure of families has changed, and with nuclear families becoming the norm, parents are responsible for all care-related activities. Therefore, the need for nurturing emotional bonding and the idea of the family as a unit for care were incorporated into the intervention modules.

##### Learnings

The concept of early child cognitive development initiation was incorporated in the App module. The parents, as well as the grandparents, have been depicted as important partners in the development journey of the child. The content of the modules, as well as the App, incorporated popular forms of engagement with the child such as pointing out daily objects, birds, and singing to the child in easy and common local songs. The aspirations that the parents had for their children were shown as achievable through early interventions along with sustained involvement and encouragement of the child in all aspects. Safety, health and nutrition were retained as an important part of the communication products, as these aspects were an existing part of the ICDS intervention, and are an essential component of the care paradigm as understood by the parents.

##### Fathering as “supplementary” and maternal gatekeeping

The fathers were viewed as helpers and subsidiary fillers in providing care for young babies, and not as responsible caregivers. This perception prevailed among fathers, mothers, other community members as well as the AWWs themselves. These beliefs stem from established societal beliefs that women are more sensitive toward children and instinctively better at taking care of them.

“*I would say that I am only helping; in general, mothers have the most interaction with the child, and so fathers help when possible. We help by playing with the child or taking them out, but mothers take care of all other needs, since we are stressed with work duties.” (Father)*

Fathers in the study sample mentioned that they were not taught to take care of children, as it is not a topic discussed between men, whereas their wives often receive advice from their mothers. They also believe mothers are natural at child-care and will be able to handle most situations more appropriately. The mother's breastfeeding role was also seen as imparting them with abilities to be the primary natural caregiver.

“*I would say women know how to raise kids and can do it in a much better way because their mothers teach them these skills. We are not used to this, so we should let them handle it.” (Father) “In terms of child-care the mother's involvement is more important than the fathers. Us men are not taught how to engage in child-care, and mothers will naturally have these skills. By nature, we are limited.” (Father)*

The fathers thus had poor confidence in their ability to take care of their babies. They also felt their child would not respond well to them even if they attempt to provide care.

“*Mothers start taking care of the baby as soon as it is born and get used to handling it, but for us, it will take a minimum of one month to start handling the baby. When they are too young, it is difficult to carry the child because we worry they may get hurt.” (Father)*

Mothers attend to the child's needs, such as feeding, bathing, cleaning, putting to sleep, talking, and playing with the child. They also take the child to the AWC for growth monitoring and to collect nutritional supplements.

##### Learnings

There was a need to address maternal gate-keeping attitudes, fathers' lack of confidence, as well as the community workers' own perceptions regarding gendered roles. Consequently, all of these aspects were addressed in the project in order to bring about a sustainable change. For the AWW trainings, the workshop modules addressed gender roles of providing care for young babies, mothers' need for rest after household chores as being equally important to fathers need for rest after paid work, and the need for exclusive time for engaging in breastfeeding as an important precursor for child health and development.

For the mothers, there was a need to engage them as a facilitator of fathers' involvement, which would in turn ensure that her gate-keeping role was reduced, and as a documenter of change, again to facilitate their involvement and nurture a sense of ownership in the project. The mothers were engaged by the AWW in an initial outreach campaign. The mothers' calendar was designed to be filled by the mothers, not only as a record of fathers' involvement, but also as a cue to action for fathers and mothers as facilitators. The calendars were to be collected every month by the AWW, and this provided the AWW an opportunity to enquire about the participation of both parents.

Self-efficacy and confidence in handling children also emerged as a critical finding from various fathers' responses about child-care practices in the household. Men felt under-confident about understanding and responding to child needs. This was especially the case when the child was distressed.

“*When my baby cries for no reason, I hesitate and do not know what to do. My wife handles him better than me because she knows why he is crying, and immediately after she picks up the child, he stops crying.” (Father)*

This important aspect was added to both the implementation strategy as well as the evaluation trial, as an essential indicator for evaluation. Fathers also mentioned that while they understand the need for their involvement in their child's care, they do not know how to get involved. They asked for practical tips on how they can aid in their child's development, and therefore, these were introduced in the modules. Suggestions were designed to be simple, cost-effective and doable using household products. For example, toy making was one of the activities in the module, and in some of the modules, the fathers were asked to bring their baby along for play sessions. The pictorial representation in the Arivu app showcased fathers involved in easy-to-do household chores, like hanging the clothes to dry, as well as those in which there were close interactions with the child, such as feeding them and involving in games.

#### Secondary Data Analysis

The secondary data sets available from the ICDS Department not only aided planning for both implementation and the research trial, but also guided the development of the training modules and materials.

##### Development of the communication materials

The communication products currently in use by the Department including posters, pamphlets, and stickers helped to ensure that all intervention material was congruent to the messaging and ideas of the state and were appropriate for the local context. The material provided had detailed meal plans in locally available food groups and songs in the local language, which were added to the communications modules during the development of the App. The final contents had additional content on father involvement and early childhood development added to the theme areas of the government communication.

##### The sampling design and adaptations

The manual records at the AWC were used as a guide to develop our sampling plan and intervention budget. We conducted house listing and pre-survey exercises to generate the actual sampling frame. This frame had fewer children compared to the manual lists, and the clusters and geography had to be doubled in order to reach the required sample of 1,400 children. This expanded the cluster sample from planned 60 to 117 AWC, from additional blocks that had to be covered by the listing and survey exercises to finalize the sampling frame. Though this meant loss of time and money, the house listing and pre-survey ensured that the primary survey team had to spend less time in identifying the children and households. Addition of clusters also increased the power of the CRT. But there were additional resource requirements, to cover the cost of the intervention.

##### The human resources planning

The Human Resources (HR) data set at the district level was useful for collecting the listing of the AWC that were manned by both a full-time AWW and a helper. From previous studies and the formative research for this study, it was found that AWWs are often overburdened with work and may find it difficult to implement the intervention without the help of the helper. Consequently, from a sampling frame of all the AWC in the selected blocks, the AWC that met the following practical and operational criteria were selected: the AWC is the main center and not a sub-center, and the center has the availability of an AWW and an Anganwadi helper. The technology augmented job-aids were designed to suit their particular requirements. Many of the workers had lower functional literacy despite the educational cutoffs for these posts. They also had limited time and patience to read and execute hard-copy manuals. Hence the tab design and the modules, designed in close coordination with ICDS and regular AWW trainers, were kept simple and largely pictorial. The design went through multiple iterations with the Department and AWW, and piloting in the community in order to ensure cultural and contextual suitability.

### Stage II

The second loop, which was essentially the operations research and process monitoring, lead to learnings and adaptations of the implementation strategies. This process was implemented by the research team, who led the formative and summative assessments, the field team, who managed the oversight of the AWW, and finally the AWWs themselves who implemented these initiatives among the participants.

#### Working With an Existing Community Worker-Led Program

During the intervention period, three out of the 58 centers had a change in AWW due to maternity leave and retirement. These centers were identified in advance, and the new AWW were provided training before they were placed. Worker retention was, therefore not a significant issue in the intervention. Worker motivation, on the other hand, was a significant hurdle. There were delays in the submission of calendars and the father's meetings in some centers. The coverage of household outreach was at around 80% and varied across months and centers.

Since the AWWs were not intervention staff, it was difficult to monitor and provide supportive supervision. There were multiple constraints and limitations faced during the deployment of the calendar and App as monitoring tools. The intervention was led by AWWs and their own performance incentives in the intervention months were linked to the calendars and the App. The incentives were kept at nominal levels to counter this, yet they may have felt pressured to report target achievements. This issue was discussed in the regular review meetings and the importance of clean data for research purposes was emphasized –yet some noise persisted in the data.

Early engagement and ownership with the government from the top down was crucial. Leadership in government played a key role in motivating the AWWs. In addition, additional cash prizes for top performers were awarded by the respective District ICDS heads in quarterly district-level review meetings where all staff from the district participated. This produced positive results as more AWWs started actively responding to the field staff over the phone. Based on the data collected, specific AWCs were targeted to improve the performances through review meetings. Sometimes due to logistical difficulties, it was not possible for the AWW to conduct the outreach at the household. Hence they were allowed to complete the same in the AWC when the parent comes to collect supplements or drop or pick up their child from the crèche.

#### Flexible Scheduling and Programming

Many fathers were unable to attend the monthly meetings as they worked out of town. Therefore, modules had to be lumped together, and a cluster of 2–3 meetings were held once every few months. At other centers, fathers expressed that Sundays were their rest days and they would prefer the meetings to be held on weekday evenings. The groups had to be split into 2–3 monthly meetings for different father groups. Clubbing modules together brought about some loss of reliability in terms of monitoring data on attendance of father's meetings being captured through the App, because it captures meetings and not modules. Splitting up meetings created additional work burden for the AWW, but on the flipside, this also eliminated issues that resulted from fathers consuming alcohol on weekends. Hence, the project ensured to implement the necessary changes to the meetings, as suggested by the fathers at each center. This allowed for increased father attendance at meetings.

“*A few of us work long days, work in different towns, or are lorry drivers who constantly travel. We usually don't even get Sundays off, and if we do that is the only day we get to rest. Because of this, we are unable to attend all the meetings and get involved with child-care to the extent that we would like.” (Father)*

Practical and instructional elements of how an intervention surrounding father involvement can work were added based on learnings from Stage I and this was well-received by AWWs and fathers.

“*We taught fathers how to make creative toys for their children using items at homes such as ribbons and dough. We would also recommend particular food items or snacks for the child. Whenever they follow one of my suggestions, they will either tell me or bring it to the next meeting to show me like excited school children.” (Anganwadi Worker)*

The use of incentives had to be altered throughout the intervention to adjust for the needs of AWWs and fathers, along with the changing mobile networks. For instance, the initial mobile-recharge based incentives had to be replaced with cash and other mediums as the mobile-based landscape in India was changing throughout the duration of the intervention. Mobile-network companies began to introduce unlimited talk time plans which deemed the recharge incentives irrelevant. Fathers also stated they prefer cash or toys in place of the phone recharge incentives so they could give back to their children.

“*This money came through my child, and so I do not want to profit from it. I believe it deserves to go to him because my main goal is to give him a better future.” (Father)*

The initial objective was that the calendars would be used to generate the incentive amounts for each father, based on their performance in the previous month. However, the challenges led to time lags—resulting in fathers receiving the incentive for the month before. Another challenge is the timely and accurate digitization required for access to the data collected. Since the calendar was a hard copy, the data entry had to be done manually, and this was cumbersome and prone to errors. However, the calendar was very popular with the mothers and aided in the monitoring process as an effective cue to action.

“*Our husbands constantly check the calendar now to see their progress and seem to be more invested in our child's brain development. My husband will continuously enquire if our child's height and weight measurement is normal, and if not what it should be. If our child's growth is below average, my husband will mention that the Anganwadi worker suggested that we buy a particular item and then will buy it the next day.” (Mother)*

#### Exclusive Father's Meetings and Incursions by Mothers

During the early stages of the intervention, AWW workers found it difficult to communicate and engage with the fathers because of hesitation and shyness on both ends. It was difficult for the AWW to control ego-clashes between the fathers in terms of scheduling meetings. A few fathers were not cooperative and vocalized their disinterest in participating in “female tasks” and would avoid attending meetings using excuses. To counter this, the team encouraged both parents to attend the initial meetings and as the intervention progressed, the fathers' attendance picked up.

“*I initially came to the meetings because I was curious and wanted to know what was being said to the fathers, but now I attend the meetings so that I can remind my husband about what he needs to do for our child and how he can help.” (Mother)*

There were modules where both parents had to participate; the clubbing of modules led to many meetings requiring both parents to attend. The team tried to convince the mothers to come to meetings only when required so that fathers did not feel that the meeting was crowded by women. Some mothers attended the meetings to fill in for the father when they were unavailable. Others accompanied the fathers to encourage their participation. Consequently, some of the exclusive fathers' meetings had some mothers participating.

Overall, the mother's role had positive influences on the intervention. Mothers encouraged the fathers to attend meetings, and monitored this using the calendars. In some cases, they overshadowed the fathers in the meetings with their enthusiasm. Yet, the mothers were a support to the AWWs to network with fathers, and to conduct the meetings and improve attendance.

#### Some Learnings Did Not Channel Change

Not all learnings could be channeled to bring about change, such as cultural practices and consumption of alcohol. In the local culture, new mothers spend approximately the first 3 to 5 months at their maternal home in order to receive help with motherhood and child-care. This is an impediment in the way of early father-child bonding. As a consequence, when the mothers returned home, fathers did not have the confidence to handle their child, and believed that the mothers were more competent and should, therefore, care for the baby. This practice was limited to some households and also applied only to first pregnancies; yet, there was an intractable loss of time for very young babies.

Alcoholism was another major problem in the blocks. In some of the villages in particular, during most holidays the men would start drinking early in the morning and would either be absent from the meetings or would come to the meetings in an inebriated state. The AWWs also reported that it was often difficult to conduct the meetings on workday evenings as some of the fathers went out for drinking in the evenings. This challenge persisted throughout the intervention year, but was worse during festivals. After speaking with the fathers' multiple times, some fathers did start attending meetings in better states.

“*Meetings are usually held on Sundays, which is when most men in the village drink. Fathers will both drink and skip the meeting entirely or, if they decide to attend the meeting, they will be a complete nuisance and distract everyone. They have no discipline or patience when they are in that state, making it a struggle for us to conduct the father meetings.” (Anganwadi worker)*

The fathers tend to drink during their limited time at home, and thus reduced the amount of time available for bonding with the child. Mothers were also wary of fathers handling the children when they were intoxicated because they were anxious about the safety of the child. Alcohol consumption affected not only the time available for child-care, but also the father's attitude toward child-care and their self-confidence in engaging with the child. This was a persistent challenge in the intervention.

“*When my husband is drunk, he does not want to handle the children as he is not confident, and I am also not comfortable with it.” (Mother)*

Another challenge was posed by unreliable phone networks which disrupted communication with fathers and the disbursal of incentives. The closure of a mobile network operator, affected around 25% of the users in the treatment group, who stopped receiving the mobile messages. It took about a month to collect the fathers' new mobile numbers information as many fathers took time transitioning to new networks. Another issue was poor network connectivity in rural areas. Per SMS sent, only about 75–90% were received by the fathers. On phone-call backchecks, it was discovered that the fathers working in remote work locations on farms, quarries and similar locations had poor network connections, or kept their phones switched off to conserve charge. Fathers, especially who were less literate, did not read messages or found it difficult to differentiate these messages from advertisements. After initial runs, the intervention therefore adopted a strategy of scheduling timings and informing fathers about when to expect messages as well as how these messages look.

### Stage III

While quantitative measures may be appropriate for measuring child outcomes, the identification of norms and norm change is best depicted through qualitative analyses. The empirical evidence from the CRT is being described in another article, which brings out the modest yet significant impact, in child development outcomes, among the children in the intervention group.

**Figure 2:**
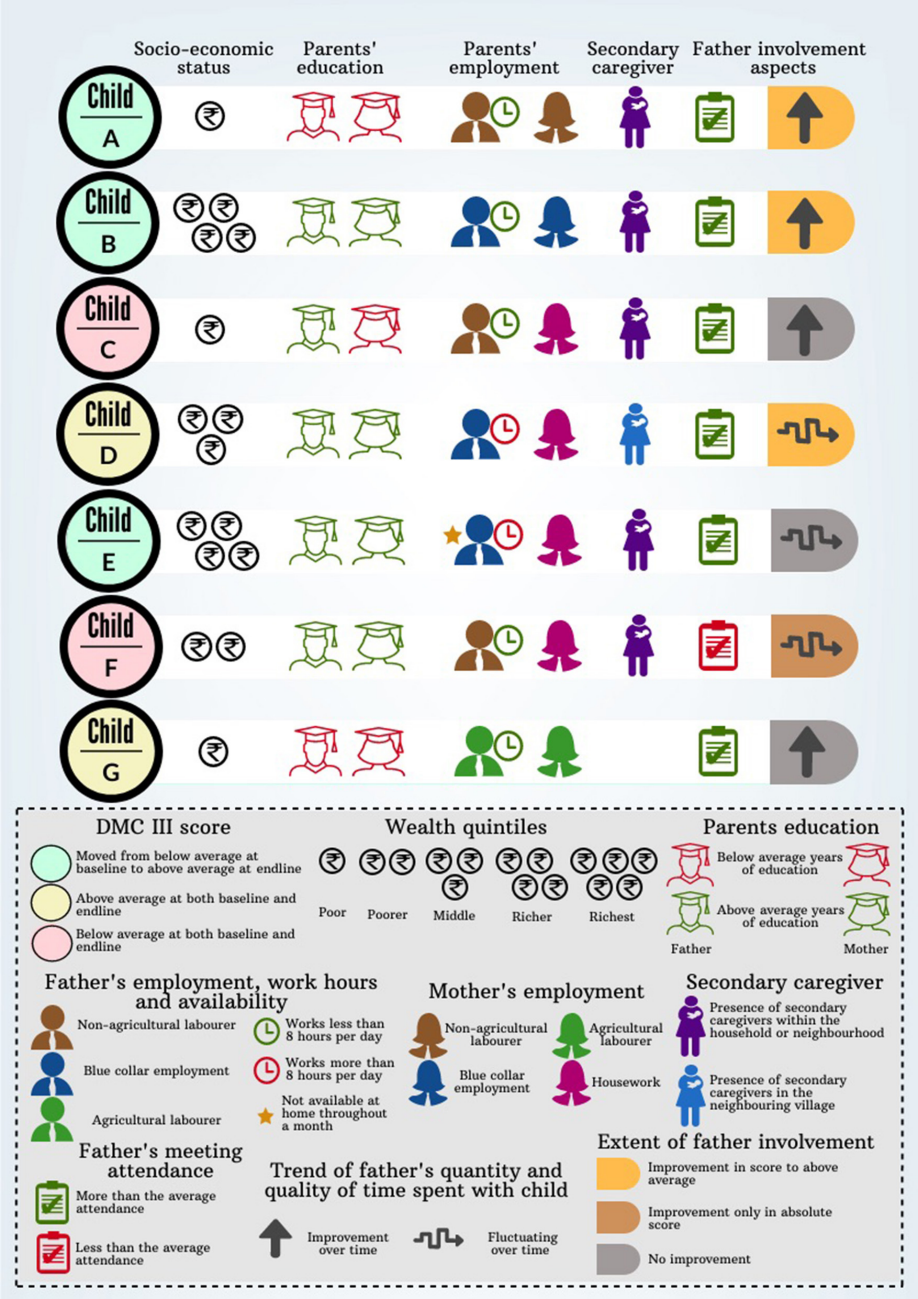
Mapping analysis from the case studies with families.

The nested qualitative longitudinal case study was used to observe changes in the family environment, in order to understand changes in time spent by parents with the child. From the data available from seven families, their SES, education, job profile of parents, availability of care support, and aspects of father involvement were interpreted against the child development gains of the child (Figure 2). The learnings brought out complex interdependencies of factors in individual child and family trajectories toward their path in child development goals.

#### Interpretation

Variables like socio-economic status, parents' education, presence of supportive caregivers, parents' occupation and availability for child-care, along with intermediate outcome variables like father involvement and dietary diversity, determine child growth in terms of DMC III score and anthropometric indicators. Since these variables are all interrelated, they collectively impact the growth of a child.

Children who have grandparents providing supportive care tend to have better developmental outcomes, despite the unavailability of the father, as in the case of Child E. Socio-economic status mattered only in terms of mother's availability of time and her burden of caregiving. For instance, more-than-average educated mothers of children D, E, and F stayed home, while less-than-average educated mothers from low-income families like that of children G and E were employed as manual laborers. Moreover, enhanced engagement of fathers and involvement of grandparents can mitigate the shortcomings of poor socio-economic status, as noticed in child A where stunting was overcome. It was observed that in most children who were fed more than the minimum variety of food items, had no evidence of stunting, being underweight, and wasting, as noticed in children B, C, and D. However, children G and C, who were fed the required variety of food but were deprived of father involvement, educated mother and wealth, fared poorly in developmental milestones. Further, parents' involvement in child-care was important for growth, more so than their level of education, as observed from both the cases of children A and G.

These findings clearly depict how fathers' involvement is multi-factorial and multidimensional with several intertwining factors like parental relationship, physical presence, residential/non-residential fathers and socio-economic factors that might act as determinants in the final outcomes related to a child's development. Behavior change interventions work primarily through changes in perceptions and knowledge, which channel through these existing determinants.

The phenomenon of father involvement, as it changed during the intervention year, can be explained by its three aspects, namely interaction, availability and responsibility. In multiple scenarios, it was observed that the intervention had enhanced the behavior of fathers toward their wards. These were the findings from the endline interactions with parents and AWW.

#### Availability

As per the advice of the AWW, fathers started to observe their children more and have learned the correct way to behave with them. Fathers are more comfortable with their child now as they have more confidence in handling varied situations.

“*Though it was initially a struggle for the fathers to attend the meetings, it started to become a routine for them, and they would attend every weekend because they became used to it. Now they are genuinely interested in the information presented as they realized it is benefiting none other than their own children.” (Anganwadi worker)*

In multiple scenarios, it was observed that the intervention had enhanced the behavior of fathers toward their wards. Despite working in manual-labor intensive positions such as the agriculture field or factory industry, where the hours are long and breaks are minimal, fathers have begun to spend time with their children during their breaks—even if they are tired after work. Some fathers even set aside an entire day once a week, during their off days, for their children.

#### Responsibility

Both parents were supportive of their children and made efforts toward rectifying the mistakes they believed their own parents made. Fathers spoke about wanting to give their children a better future and provide them with opportunities not available to them. Fathers are also very involved in checking their progress as well as their child's growth and development.

“*I used to think that children are a mother's responsibility because that is how it was when I was growing up. But now through the meetings and messages, I know that men are equally responsible.” (Father)*

The fathers regularly bring their child to the AWC for their height and weight measurement check-ups and are interested in comparing their child's improvement from the previous month. Through the course of the intervention, both mothers and fathers perceived the importance of cultivating paternal relationships in early childhood, and the prominent role fathers play in a child's life.

#### Interactions

Fathers also tended to teach their children educational lessons such as colors or the alphabet and help them with their drawings when they notice their children attempting to grab pens and scribble. This is an improvement to the previous situation where fathers would only play cellular-phone games with their children. Once fathers realized they have a direct impact on their child's well-being, fathers took household tasks and child-care duties more seriously. They have begun to take their children out on walks, play with them, feed them, and even dress/bathe them if needed. At the end of the intervention, it was noticed that there was also an increase in engagement between AWWs and fathers, as fathers began to seek suggestions and be responsive in meetings.

“*We can see a clear difference in the behavior of our husbands and children now, compared to before. Our husbands were not as caring before the intervention, but now they spend more time with the children and have learnt how to take care of them appropriately. Our children even seem happier and more active because of this.” (Mother)*

Without exposure to examples of paternal involvement in their childhood, the fathers struggled to grasp this concept or gave up completely. Some fathers, despite aiming to be more involved, seemed to select relatively easier, enjoyable tasks such as taking the child out on walks. The families and AWWs appreciated the concept of the intervention and felt the combination of tablet usage, meetings and calendars benefitted the children. Mothers and children tend to enjoy their time at the center, and mothers questioned why the program could not be extended, as they believed it helped both them and their children learn.

## Discussion

This was a Proof of Concept study, and therefore the main objective was to ensure that the learning process continues through the period of the study, as well as obtain evidence to inform future model development. The formative assessment deepened our understanding of the community as well as its prevailing norms and helped identify the factors and facilitators of change. Emergent learnings from the field were used to innovate and problem-solve to overcome a challenge or to improve the strategy in a consensual manner with discussions with the staff and inputs from the community. Flexibility in outreach strategy, integration of norm changes into the existing framework of parenting, linkage to the existing public project ensured that despite conflicting with traditional gender roles, parents show evidence of developing positive attitudes to fathers' involvement in child care and early initiation in cognitive development.

The final learnings were then captured in order to be available for future model development. Thus, this study implemented the Measurement for Change principles of heterogeneity, evidence, and interaction. The learnings through the MEL in the intervention generated valuable learnings with regards to fathers' involvement in child-care in rural community settings. Some of the overall learnings are as follows:

### Listening to Parents' and Providers

The objective was not only educating the fathers on paternal involvement, but to facilitate norm change. Therefore, it was essential that the project not only generated an understanding of the current norms, factors, and facilitators, but also ensured that the inputs to strategy worked to integrate norm change into the existing framework of parenting as understood by the communities. Similarly, it was essential for the model to be able to integrate into the existing public welfare projects, and for the learnings to be easily incorporated into the existing behavior change communication framework.

### Expanding the Domain of Care and Making It Practical

In the communities, parents often sit with their child but were watching over them to ensure safety rather than interacting with them. From this and previous studies, it can be determined that there is insufficient knowledge about stimulation and child development (18). This was a novel component and was received positively by the parents. Despite the difficulties in transitioning from their traditional gender roles, the fathers were willing to change norms because they perceived it to be beneficial to their child's development. Engaging with the child became easier with the practical tips provided through the project. Ensuring safety, health, and nutrition were also the biggest domains of care for children of this age group, and therefore continued to be the focus of the communication with parents.

### Mothers as Important Facilitators and Motivators for Father Involvement

The importance of mothers as the drivers of change in the child development space in rural settings was a key learning. Though mothers initially served as barriers and gatekeepers in the way of father-child bonding, they eventually became enablers in the process. The new parenting environment affects the conventional family structure, and it is important to address any skepticism among mothers about fathers assisting with child-care. Mixed parent groups may elicit higher trust and experiments elsewhere have found couple groups to be effective (19). Mothers are critical decision-makers in the life of young babies and also have a close rapport with the female community workers. Therefore, interventions need to rope in their support from the very beginning.

### Flexible Engagement Strategies

Incentives, particularly phone recharges, are useful to establish meaningful connections with fathers and need to be presented in a way that is enabling and engaging. For example, keeping the incentive low and tying it to time spent with children was both acceptable as well as motivating. There should also be flexibility regarding the way in which such performance-based incentives are given. Phone recharges may be difficult in circumstances where mobile phone companies or plans and tariffs change often, or rural poor may shift priorities or may use it intermittently owing to travel and other factors. Therefore, to ensure continued engagement payment modality may be required to change to cash or both. Messaging given through phones need to be timed to be received at convenient points and intervals and may need reminders. Similarly, meetings may need to be flexibly scheduled to suit the work-life balance of the fathers, including bundling modules, evening meetings, using weekends or splitting batches.

### Working With the System to Ensure Sustainability and Scalability

To ensure scalability of such interventions, it is crucial to work with the largest provider of care in the setting, the public provider. The project worked with the Government, keeping them engaged at the state district and block levels from the very beginning. A sense of ownership was built from the planning stages through incorporating inputs in the materials produced, and ensuring congruence with the existing communication materials. This was continued through the implementation by involvement measures, and reporting during the ongoing training and monitoring. There are inherent challenges in ensuring efficiencies when working with frontline workers who are over-burdened and under-motivated, and these have been documented by many other interventions (20, 21). The project was successful in overcoming this to an extent through planning and supportive supervision.

Through the course of the intervention, there was evidence of a gradual shift in the mind-set of the parents and community workers to view the fathers from being solely providers to an involved and caring parent responsible for their child's development. This was an important yet small beginning in the trajectory of behavior change. Study limitations include a lack of time during the qualitative endline period as it extended beyond expected timelines, and elections hampered collection of monitoring data. One year of intervention is a short time for effectively bringing about lasting behavior change in a community. However, the study results suggest that it did bring an incremental change in attitudes, which is a valuable initial step to change and progression.

Many questions remain unanswered for scaling up child development interventions in rural settings in India. While there has been policy and programmatic direction toward digitization, the AWW remains a low-wage, high-burden, poor-motivation, low-social capital workforce. It may be challenging to engender changes in norms through AWW alone. Role model fathers may have to play a more significant role in many settings, especially where the patriarchal structures are entrenched. Alcoholism may worsen the effect, and models with additional male helpers may be practical. Structural challenges like poverty, illiteracy, and migration are hurdles in outreach, especially among fathers. Structural challenges also deprioritize child development within the family due to their other pressing requirements. A rapid expansion in the use of mobile phones, especially among men, and increased use of digital payments has favored interventions using phone-based incentives. Network plans and connectivity issues, and the rapid changes of providers are potential hurdles in these models. There is also a fundamental question of incentivizing a behavior, that is essentially a duty. This can be overcome by the argument of compensating parents' for their loss of wages or time, and has been employed in the form of cash (22), material (23), and mobile talk time (24) in low-income settings.

Along with the successes and the challenges, the MEL system also examined how fathers' involvement strategies can be improved through the continuous embedded process of monitoring, evaluation and learning. The paper discusses tweaks in the structural elements in an existing framework for community outreach in child development, and how this can be useful to involve fathers in a feasible manner. These results will be beneficial in refining future models, scaling in other states, as well as informing policy direction.

## Data Availability Statement

The raw data supporting the conclusions of this article will be made available by the authors, without undue reservation.

## Ethics Statement

The studies involving human participants were reviewed and approved by IFMR Human Subjects Committee (dated September 26, 2017). Written informed consent was provided by all participants, the parents in the study.

## Author Contributions

SN is the principal investigator of the study and was involved in the study planning, training, data monitoring and analysis, and writing the manuscript. SC and NS coordinated the study data management, analysis, and write up. RS and AR were involved in the study design and implementation. All authors contributed to the article and approved the submitted version.

## Conflict of Interest

The authors declare that the research was conducted in the absence of any commercial or financial relationships that could be construed as a potential conflict of interest.

## Abbreviations

AWW(s): Anganwadi Worker(s), government-based child-care community workers
AWC: Anganwadi Center: public crèches run by Anganwadi Workers
ICDS: Integrated Child Development Service: flagship child development scheme of the Government of India
MEL: Monitoring, Evaluation, Learning.
